# Measuring the Frequency of Inner-Experience Characteristics by Self-Report: The Nevada Inner Experience Questionnaire

**DOI:** 10.3389/fpsyg.2018.02615

**Published:** 2019-01-11

**Authors:** Christopher L. Heavey, Stefanie A. Moynihan, Vincent P. Brouwers, Leiszle Lapping-Carr, Alek E. Krumm, Jason M. Kelsey, Dio K. Turner, Russell T. Hurlburt

**Affiliations:** Department of Psychology, University of Nevada, Las Vegas, Las Vegas, NV, United States

**Keywords:** inner experience, questionnaire, descriptive experience sampling, inner speech, inner seeing, unsymbolized thinking, feelings, sensory awareness

## Abstract

Descriptive experience sampling has suggested that there are five frequently occurring phenomena of inner experience: inner speaking, inner seeing, unsymbolized thinking, feelings, and sensory awareness. Descriptive experience sampling is a labor- and skill-intensive procedure, so it would be desirable to estimate the frequency of these phenomena by questionnaire. However, appropriate questionnaires either do not exist or have substantial limitations. We therefore created the Nevada Inner Experience Questionnaire (NIEQ), with five subscales estimating the frequency of each of the frequent phenomena, and examine here its psychometric adequacy. Exploratory factor analysis produced four of the expected factors (inner speaking, inner seeing, unsymbolized thinking, feelings) but did not produce a sensory awareness factor. Confirmatory factor analysis validated the five-factor model. The correlation between an existing self-talk questionnaire (Brinthaupt’s Self-Talk Scale) and the NIEQ inner speaking subscale provides one piece of concurrent validation.

## Introduction

The term *inner experience* as we will use it here refers to directly apprehended “before the footlights of consciousness” inner events such as inner speaking, visual images, and sensations. *Pristine* inner experience refers to inner experiences in their natural state, undisturbed by the act of apprehension, not manipulated by psychological experiment or any other specific intervention (Hurlburt and Akhter, 2006; Hurlburt, 2011).

Descriptive experience sampling (DES; Hurlburt, 1990, 1993, 2011; Hurlburt and Heavey, 2002, 2006; Hurlburt and Akhter, 2006) is an explorational method aimed at pristine inner experience. It uses a random beeper and “expositional” interviews to investigate instances of pristine inner experience. Of course, it falls short—the beep and its response requirements by definition disturb the pristine nature of the experience. Therefore, the aim of DES is to get a glimpse of pristine inner experience in as high fidelity as the current state-of-the-art allows.

The DES method has been described in detail elsewhere (Hurlburt and Heavey, 2006; 2017; Hurlburt, 2011, 2017), and its methodological adequacy has been discussed (Hurlburt and Schwitzgebel, 2007; Caracciolo and Hurlburt, 2016; all the papers in Weisberg, 2011).

Heavey and Hurlburt (2008) have said that there are five frequent phenomena (subsequently dubbed the “5FP” by Kühn et al., 2014) of inner experience: inner speaking (sometimes called “inner speech”; Hurlburt et al., 2013), inner seeing (sometimes called “visual imagery”; Hurlburt, 2011), unsymbolized thinking (a thought directly present without words, images, or other symbols; Hurlburt and Akhter, 2008a,b), feeling (the experience of emotion; Heavey et al., 2012), and sensory awareness (attending to some sensory aspect of the internal or external environment without regard for instrumentality; Hurlburt et al., 2009). Each of the five occurs in roughly a quarter or more of samples (adding to more than 1 because several features can occur simultaneously). To say something like “a characteristic occurs a quarter of the time” implies the necessity of measuring the frequency of these characteristics. Heavey and Hurlburt (2008) measured the 5FP frequencies in the scientifically standard way: they used DES to obtain random samples of inner experience, counted the number of those samples that contain the characteristic and divided by the total number of samples.

Descriptive experience sampling is a labor-intensive procedure, so it would be desirable, if possible, to have a more efficient way of estimating frequency of the 5FP, such as by questionnaire. However, no such questionnaires exist. There are two questionnaires that consider the frequency of inner speech: the Self-Talk Scale (STS: Brinthaupt et al., 2009) and the Varieties of Inner Speech Questionnaire (VISQ; McCarthy-Jones and Fernyhough, 2011; and the revised version VISQ-R, Alderson-Day et al., 2018). The STS has two frequency-related limitations. First, it does not inquire directly about frequency in natural settings. Instead, the STS inquires about frequency in specific situations, by presenting the stem “I talk to myself when…” followed by a list of situations such as “I should have done something differently,” or “I want to reinforce myself for doing well” (Brinthaupt et al., 2009, p. 88). There is no measure of how frequent those situations are and therefore no way of translating to overall natural-setting frequency. Second, it uses anchors (1 = *Never*, 2 = *Seldom*, 3 = *Sometimes*, 4 = *Often*, and 5 = *Very Often*) that are ambiguous: “Often” might refer to five times a day (“I *often* brush my teeth”) or five times a year (“Hurricanes *often* make landfall in the US”). Despite these limitations, the STS is occasionally used as an overall frequency measure (Brinthaupt et al., 2015) by recoding the ratings from 0 to 4 instead of 1 to 5, adding them, and dividing by 64 (the possible sum of scores), a procedure that assumes (with little warrant) equality of frequency across situations and across people.

The VISQ (McCarthy-Jones and Fernyhough, 2011) is a questionnaire designed to measure features of inner speech inspired by Vygotsky. Like the STS, it has two frequency-related limitations. First, instead of inquiring about frequency directly, it asks about Vygotsky-inspired characteristics of inner speech. Second, it uses ambiguous anchors (1 = *Certainly does not apply to me*, 2 = *Possibly does not apply to me*, 3 = *If anything, slightly does not apply to me*, 4 = *If anything, applies to me slightly*, 5 = *Possibly applies to me*, and 6 = *Certainly applies to me*), which are not really measures of frequency at all. Here is a typical item: “I hear the voice of another person in my head. For example, when I have done something foolish I hear my mother’s voice criticizing me in my mind” (McCarthy-Jones and Fernyhough, 2011, p. 1589); there is no measure of how frequent “doing something foolish” is, and no direct way of mapping *applies to me* onto frequency. The recently revised version (VISQ-R, Alderson-Day et al., 2018) reduces the anchor ambiguity by using as anchors 1 = *Never* to 7 = *All the time*, but the VISQ-R remains a consideration of the characteristics of inner speech when it occurs, not a measure of its frequency of occurrence.

There are questionnaires inquiring about emotion (e.g., the Positive and Negative Affect Scale; PANAS; Watson et al., 1988), but such questionnaires typically rate the *intensity* of emotion, not the frequency of feelings. There are questionnaires inquiring about visual imagery (e.g., the Vividness of Visual Imagery Questionnaire; VVIQ; Marks, 1973), but those questionnaires typically rate *vividness* of imagery, not its frequency. There are, that we know of, no questionnaire measures at all for unsymbolized thinking or sensory awareness as DES defines them.

Many psychologists believe that inner experience is important for both theoretical and practical reasons. Using inner speech as an example, theoretically, Baddeley and Jarrold (2007) held that inner speech instances are recitations in a phonological loop designed to keep information readily at hand. Practically, inner speech is held to be important, for example, in a wide variety of sport (basketball, football, golf, tennis, cricket, cross country running, swimming, volleyball and many others) performance (Hardy, 2006; Van Raalte et al., 2014), in psychotherapy (Meichenbaum, 1977), in self-awareness and metacognition (Morin, 2005, 2011; Carruthers, 2011), and so on. However, claims about the frequency of inner speech vary widely, from “Human beings talk to themselves every moment of the waking day” (Baars, 2003, p. 106) to the 28% found by Heavey and Hurlburt (2008). Any theory about the role of inner speech in information processing, sport success, psychotherapy, and so on must account for or dismiss claims about individual differences in inner speech frequency (Hurlburt et al., 2013).

Thus, inner experience (including inner speech) is important, and the measurement of the frequency of inner experiences is a basic scientific endeavor. DES is the best method we know of for such frequency measurement; however, DES is time intensive, so it would be desirable to estimate frequencies by questionnaire. Current questionnaires, if they exist at all for inner phenomena, typically measure characteristics such as vividness rather than frequency, and their response anchors are often ambiguous.

**Table 1:** The Nevada Inner Experience Questionnaire (NIEQ).

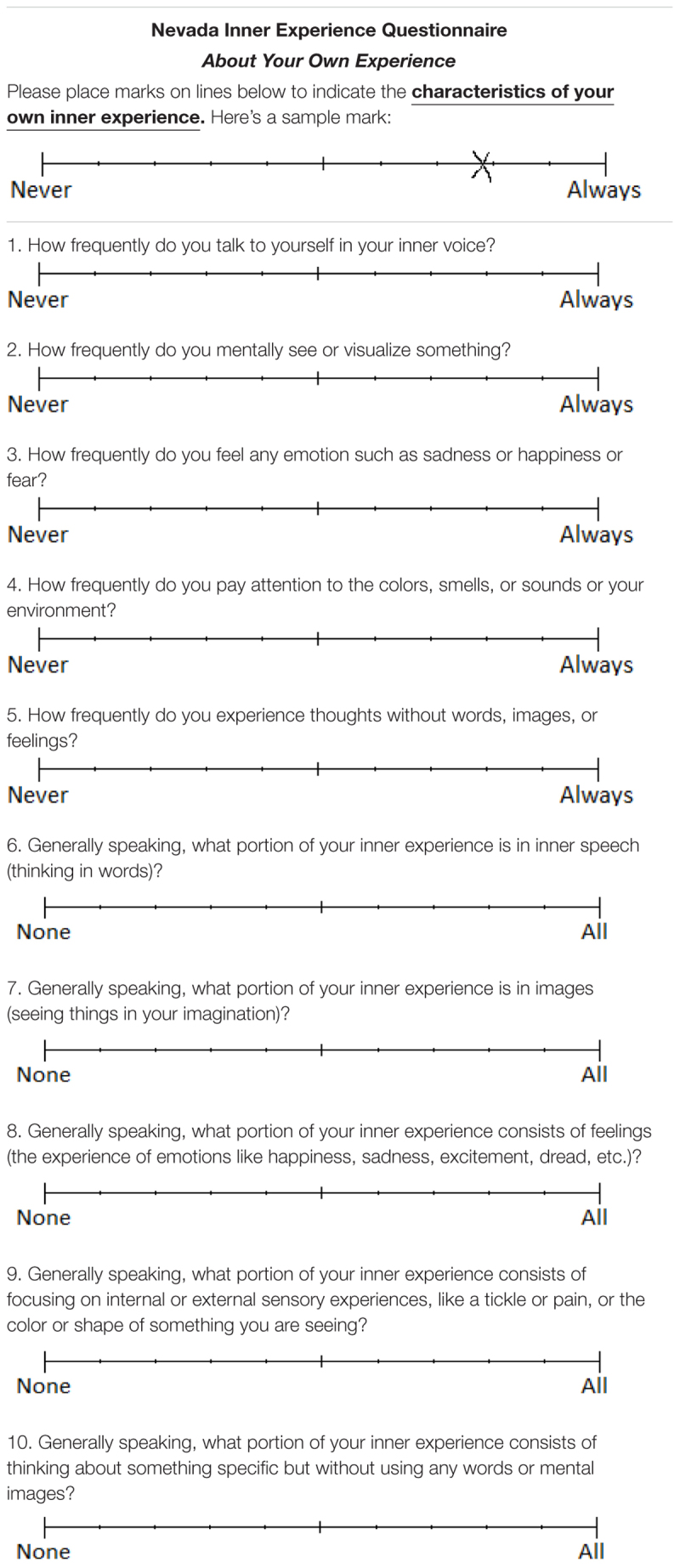

To overcome all those limitations, we created a questionnaire (the Nevada Inner Experience Questionnaire; NIEQ) that (a) inquires about the same inner phenomena that DES frequently finds (the NIEQ has five subscales, one for each of the 5FP); (b) inquires directly about the frequency of experience, rather than its vividness, etc. (by asking “How frequently do you…?” and “Generally speaking, what portion of your inner experience is…?”); (c) inquires about frequency in the natural environment (not about a specified list of situations or a specified list of characteristics); and (d) reduces the ambiguity of anchors by using visual-analog scales (Wewers and Lowe, 1990) with anchors from *Never* to *Always* (for the “How frequently do you…?” questions) or from *None* to *All* (for the “Generally speaking, what portion of your inner experience is…?” questions). The complete NIEQ is shown in Table 1. The present study investigates the psychometric adequacy of the NIEQ.

## Materials and Methods

### Participants

The participants were undergraduate subject-pool volunteers (*N* = 260) taking introductory psychology courses at a large urban university. It was a diverse sample: mean age = 20.6 years (*SD* = 4.35; range = 18–49); 28.5% male, 63.5% female, 8% did not provide gender information; 39% self-identified as white or Caucasian, 17% Hispanic, 15% African American, 15% Asian, and 8% Pacific Islander. Each received subject-pool credits for participation.

**Table 2:** NIEQ item and scale means (and standard deviations), percentages^a^, and STS score and percentage.

Item	ISpeaking	ISeeing	UnsTh	Feeling	SensAw	STS Score	STS percentage^b^
*Frequently*	70.7^c^ (22.1)	71.0 (24.0)	40.6 (29.5)	79.2 (19.0)	72.8 (21.4)		
*Generally*	65.9 (19.9)	61.2 (24.0)	35.0 (25.7)	69.4 (22.5)	51.0 (23.7)		
Scale^d^	68.3 (17.1)	66.1 (20.7)	37.8 (23.4)	74.3 (17.9)	61.9 (17.5)	59.0 (9.9)	67.2 (15.4)


*ISpeaking, inner speaking; ISeeing, inner seeing; UnsTh, unsymbolized thinking; SensAw, sensory awareness. ^a^N = 260. ^b^Derived from the STS Score following Brinthaupt et al. (2015): STS percentage = 100 × (STS total – 16)/64. ^c^Participants’ responses on each NIEQ item ranged from 0 to 100% except Feeling/Frequently (range 9-100%) and Unsymbolized/Generally (range 0-98%). ^d^Scale score = average of Frequently item and Generally item.*

**Table 3:** NIEQ item correlations^a^.

		Frequently				Generally				
		ISeeing	UnsTh	Feeling	SensAw	ISpeaking	ISeeing	UnsTh	Feeling	SensAw
*Frequently*	ISpeaking	0.37	-0.05	0.20	0.13	0.33	0.26	-0.08	0.22	0.14
	ISeeing		0.01	0.14	0.33	0.10	0.49	-0.01	0.16	0.18
	UnsTh			0.00	0.21	-0.07	0.01	0.43	0.05	0.16
	Feeling				0.20	0.11	0.22	-0.03	0.48	0.22
	SensAw					-0.14	0.31	0.14	0.20	0.21
*Generally*	ISpeaking						0.08	-0.17	0.05	0.06
	ISeeing							0.07	0.29	0.34
	UnsTh								0.10	0.32
	Feeling									0.31


*ISpeaking, inner speaking; ISeeing, inner seeing; UnsTh, unsymbolized thinking; SensAw, sensory awareness. ^a^df = 258.*

### Instruments

#### The Self-Talk Scale (STS; Brinthaupt et al., 2009)

The STS is a 16-item questionnaire that uses 5-point frequency scales (1 = *Never*, 5 = *Very Often*) to ask about the frequency of self-talk in various situations. It thus produces a total score between 16 and 80. Brinthaupt et al. (2009) showed that the STS has adequate test-retest reliability [*r*(99) = 0.66, *p* < 0.001] over a 3-month period. The STS defines self-talk as including either aloud self-talk or inner speech, without differentiating the two.

#### Nevada Inner Experience Questionnaire (NIEQ)

The NIEQ is a 10-item set of visual-analog scales with one pair of items (a *Frequently* item and a *Generally* item) for each of the 5FP. The scale items were written collaboratively by a team of researchers familiar with DES. One question (“How frequently…?”) was aimed at the participant’s perception of how frequently they experience the phenomenon without regard for any other phenomena, whereas the other question (“Generally speaking, what portion…?”) used softer language to evoke the participant’s perception of how frequently they experience the phenomenon, with an appreciation for time spent engaged in other phenomena. Thus, the two items of each pair were designed to ask basically the same question in two different ways. For example, the two inner speech items are “How frequently do you talk to yourself in your inner voice?” rated on a visual analog scale from *Never* to *Always*; and “Generally speaking, what portion of your inner experience is in inner speech (thinking in words)?” rated on a visual analog scale from *None* to *All*. The complete NIEQ questionnaire is shown in Table 1. The visual analog scales were treated as running from 0 to 100. Measurement was double-entry (Barchard and Pace, 2011): Two raters independently measured each rating (for example, the “sample” mark in Table 1 would be measured as 78). The correlation between raters was >0.99 for each item. Where between-rater ratings differed by 3 or more, two independent judges resolved the discrepancy. The rating for each item was entered as the average of the two raters. Ratings for each item pair were averaged to produce subscale scores for the frequencies of inner speaking (averaging items 1 and 6), inner seeing (items 2 and 7), unsymbolized thinking (items 5 and 10), feelings (items 3 and 8), and sensory awareness (items 4 and 9).

#### A Demographic Form

Designed for this study, the form asked participants to provide name, preferred phone number, age, race/ethnicity, sex, marital status, education level, and employment.

### Procedure

After obtaining informed consent, participants were administered the STS, NIEQ, and the demographic form. This took approximately 20 min.

## Results

The NIEQ item and scale means (as percentages) and standard deviations are shown in Table 2. As expected, within each phenomenon (inner speaking, inner seeing, etc.), the *Frequently* and *Generally* item pairs had similar means (with the possible exception of sensory awareness). For example, the ISpeak subscale suggests that our participants believed that inner speaking occurred on average 68.3% of the time.

Table 2 also shows the mean STS Score for our participants, as well as the STS percentage, an estimate derived (following Brinthaupt et al., 2015) from the STS Score by recoding the anchors from 0 to 4 (instead of 1 to 5), adding the new item codes, and dividing by 64 (the number of items × 4, the maximum score for each item). Thus, on the STS our participants reported self-talk (including both inner speech and external self-speech) as occurring in 67.2% of potential situations, a value very close to their NIEQ inner-speaking percentage (68.3% of the time).

**Table 4:** Varimax rotated factor components of the NIEQ (eigenvalues > 1).

		Component			
		ISpeaking	ISeeing	UnsTh	Feeling
*Frequently*	ISpeaking	0.643	0.411	-0.056	0.140
	ISeeing	0.204	0.831	-0.037	-0.015
	UnsTh	-0.049	0.027	0.780	-0.062
	Feeling	0.057	0.093	-0.074	0.838
	SensAw	-0.307	0.661	0.186	0.179
*Generally*	ISpeaking	0.874	-0.075	-0.053	0.049
	ISeeing	0.122	0.725	0.063	0.231
	UnsTh	-0.123	0.004	0.834	0.023
	Feeling	0.053	0.150	0.095	0.821
	SensAw	0.157	0.239	0.509	0.403


*ISpeaking, inner speaking; ISeeing, inner seeing; UnsTh, unsymbolized thinking; SensAw, sensory awareness.*

The NIEQ item correlations are shown in Table 3. As expected, within each phenomenon (inner speaking, inner seeing, etc.), the *Frequently* and *Generally* item pairs correlated fairly strongly with each other (see main diagonal) and the off-pair item correlations were relatively low (with some exceptions, mostly involving sensory awareness).

Because there is no existing factor model of the NIEQ, we include the results of an exploratory factor analysis in Table 4, which shows the Varimax rotated factor components when the eigenvalues are constrained to be greater than 1. Factors emerge as expected (highest loading on the pair of *Frequently* and *Generally* item), so the respective factors are easily named Inner Speaking, Inner Seeing, Unsymbolized Thinking, and Feeling. A sensory awareness factor did not emerge; the sensory awareness items loaded on all the factors.

**Table 5:** Goodness of fit statistics for NIEQ confirmatory factor analysis (robust solutions for one- and five-factor models).

Model	CFI	RMSEA (90% CI)	AIC	S-B χ^2^
One factor	0.596	0.122 (0.103 – 0.140)	99.332	169.332 (*df* = 35; *p* < 0.001)
Five factors	0.939	0.056 (0.029 – 0.082)	-4.655	45.345 (*df* = 25; *p* = 0.008)


*CFI, Comparative Fit Index; RMSEA, Root Mean Square Error of Approximation; AIC, Akaike’s Information Criterion; S-B χ^2^, Satorra-Bentler scaled chi-square statistic.*

Because the test construction was designed around a five-factor model, we used EQS (Bentler, 2008) to conduct two confirmatory factor analyses of the NIEQ, first assuming one factor (to determine whether the NIEQ represents a general inner experience factor) and then five factors (to determine whether the NIEQ reflects the five 5FP factors as designed). Table 5 presents the confirmatory factor analysis goodness of fit statistics. Because Mardia’s coefficient for the analysis was 21.38 (that is, greater than 5.00; Bentler, 2008), the data violated assumptions of normality, so robust fit statistics are displayed. The first row of Table 5 shows that the one-factor analysis did not meet the CFI > 0.90 (Bentler, 1990) and RMSEA < 0.08 (Steiger and Lind, 1980) criteria for good fit. However, the second row shows that the five-factor model provided a much better fit (AIC = -4.655) than did the one-factor model (AIC = 99.332); the Comparative Fit Index was 0.939 and the Root Mean Square Error of Approximation was 0.056.

**FIGURE 1:**
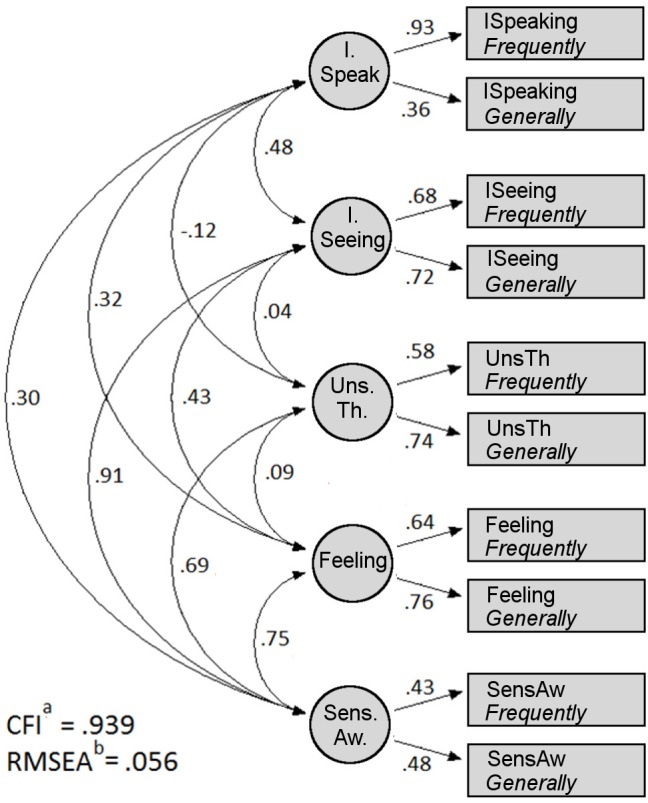
Confirmatory factor analysis of the NIEQ. ISpeaking, inner speaking; ISeeing, inner seeing; UnsTh, unsymbolized thinking; SensAw, sensory awareness. ^a^Comparative Fit Index. ^b^Root Mean Square Error of Approximation.

The confirmatory factor analysis results for the five-factor model are illustrated in Figure 1. The items typically loaded as expected: one factor was composed primarily of the inner speaking *Frequently* and *Generally* items; another primarily of the inner seeing *Frequently* and *Generally* items; and so on for each of the five factors. The weakest factor loadings (0.43 and 0.48) and strongest between-factor correlations (e.g., 0.91 with inner seeing) involved sensory awareness. Thus, the five-factor model largely (with the possible exception of sensory awareness) supports the structural validity of the NIEQ.

**Table 6:** Coefficient alpha (on main diagonal, intercorrelations^a^ of NIEQ subscales, and subscale correlation with the STS).

NIEQ Subscale						STS
Subscale	ISpeaking	ISeeing	UnsTh	Feeling	SensAw	Percentage
ISpeaking	0.50	0.30	-0.13	0.21	0.08	0.52
ISeeing		0.66	0.02	0.27	0.43	0.27
UnsTh			0.60	0.05	0.31	0.01
Feeling				0.65	0.35	0.36
SensAw					0.34	0.13


*ISpeaking, inner speaking; ISeeing, inner seeing; UnsTh, unsymbolized thinking; SensAw, sensory awareness. ^a^df = 258.*

Table 6 shows on the main diagonal coefficient alpha for each of the five NIEQ subscales; these are acceptably high for two-item scales (between 0.50 and 0.66) except for sensory awareness (0.34). The subscale intercorrelations are shown off the diagonal. Again except for sensory awareness, these are, as is desirable, relatively low.

Table 6 also shows the relatively high correlation (0.52) between the NIEQ-ISpeaking subscale and the STS percentage.

## Discussion

The NIEQ was designed to measure directly by questionnaire the five frequent phenomena (5FP) of inner experience identified by DES studies. Psychometric evaluation showed that the NIEQ behaved as it was designed: confirmatory factor analysis showed that the five-factor model was a good fit for the NIEQ items and that the items loaded in the expected way (with the possible exception of sensory awareness).

To situate the NIEQ in the context of other questionnaires, we investigated the relationship of the NIEQ-ISpeaking subscale with the STS (Brinthaupt et al., 2009), a questionnaire that has been used to estimate the frequency of self-talk. We found very similar percentages between the NIEQ-ISpeaking subscale average and the STS frequency average (68.3% vs. 67.2%) across our 260 participants; the confidence interval for the difference between the NIEQ-ISpeaking subscale and the STS included zero. [Our STS percentage was somewhat higher than the 58.6% STS percentage reported by Brinthaupt et al. (2009) and the 53.9% reported by Brinthaupt and Kang (2014); we have no explanation for this other than the samples were from different universities.] Furthermore, we found, as expected, a relatively high correlation (0.52) between the NIEQ-ISpeaking subscale and STS. The correlation should not be expected to be higher because (a) whereas the NIEQ-ISpeaking and the STS have substantial overlap (both measure inner speaking), their aims are not identical (the STS, unlike the NIEQ, also includes aloud self-talk, and the STS measures frequency in defined situations, rather than in the natural environment); and (b) there are only two NIEQ-ISpeaking items.

It would be desirable to subject the other NIEQ subscales to similar concurrent validity analysis. We did not do so because, as we have seen, such questionnaires either do not measure frequency (for imagery and feelings) or do not exist (for unsymbolized thinking and sensory awareness).

The NIEQ-SensAw subscale had lower within-scale (*Frequently* vs. *Generally*) correlation and higher between-subscale correlations than the other NIEQ subscales. We offer two potential explanations. First, sensory awareness, as DES defines it, involves a variety of sensations of both the external environment (color, smell, shape, etc.) and inner environment (tickle, soreness, stomach ache, etc.). However, the NIEQ SensAw *Frequently* item (“How frequently do you pay attention to the colors, smells, or sounds or your environment?”) inquires only about the external world, whereas the NIEQ SensAw *Generally* item (“Generally speaking, what portion of your inner experience consists of focusing on internal or external sensory experiences, like a tickle or pain, or the color or shape of something you are seeing?”) inquires about both the inner and the external world. That difference in focus might lower the between-item correlation, even though the two items together may do a better job of measuring sensory awareness as conceptualized in the 5FP than would either item alone.

Second, the concept of sensory awareness does intersect with the other 5FP. For example, feelings can importantly involve sensations (e.g., of a teary eye or a heavy heart); inner seeing may involve a specific sensory focus (e.g., on the color of what is imaginarily seen). Thus, it may be a desirable feature (not a weakness) of the NIEQ to demonstrate the correlation of sensory awareness with other aspects. Further research, including the sampling of experience in the natural environment, is required to tease apart possibilities.

**Table 7:** Varimax rotated factor components derived from Lapping-Carr (unpublished).

		Component			
		ISpeaking	ISeeing	UnsTh	Feeling
*Frequently*	ISpeaking	0.809	0.277	0.220	-0.031
	ISeeing	0.265	0.773	0.103	0.028
	UnsTh	-0.063	0.170	0.840	0.203
	Feeling	0.189	0.094	0.117	0.750
	SensAw	0.528	0.396	-0.300	0.232
*Generally*	ISpeaking	0.729	-0.254	0.108	0.419
	ISeeing	-0.149	0.877	0.238	0.203
	UnsTh	0.275	0.184	0.786	0.151
	Feeling	0.036	0.215	0.189	0.869
	SensAw	0.206	0.536	0.294	0.396


*ISpeaking, inner speaking; ISeeing, inner seeing; UnsTh, unsymbolized thinking; SensAw, sensory awareness.*

We can compare our results to those derived from Lapping-Carr (unpublished), which administered the NIEQ as part of a larger study. Those participants (*N* = 60) responded to a Qualtrics version of the NIEQ where they used the mouse to click the NIEQ visual analog scales. Table 7 shows that the results of performing the Varimax-rotated four-factor exploratory factory analysis on Lapping-Carr’s unpublished data are very similar to our own results shown in Table 4: Factors emerged as expected (highest loading on the pair of *Frequently* and *Generally* item) for Inner Speaking, Inner Seeing, Unsymbolized Thinking, and Feeling, but a sensory awareness factor did not emerge; the sensory awareness items loaded on all the factors. That is, the psychometric conclusions we drew from our own study are consonant with the Lapping-Carr (unpublished) NIEQ data.

Thus, overall we conclude that by the usual psychometric standards, the NIEQ measures the 5FP with consistent estimated frequencies and reliabilities. However, the inner experience frequencies shown in Table 2 (which ranged from 38 to 74%) are substantially higher than those reported by Heavey and Hurlburt (2008, p. 6) using DES: inner speech = 26%, inner seeing = 34%, unsymbolized thinking = 22%, feeling = 26%, and sensory awareness = 22%. These discrepancies might be due to the fact that the NIEQ, like the STS, VISQ, and other questionnaires, measures participants’ self-reports about inner experience rather than attempting to sample experience itself (Hurlburt et al., 2013). Without training and practice, participants may not have an adequate understanding of their own inner experience, so self-reports (including with the NIEQ) might be expected to over-estimate general experiential frequencies as measured by DES (Hurlburt and Heavey, 2015). We would value studies that seek to measure experience more directly, such as in the experience sampling studies by Brinthaupt et al. (2015) and in DES studies. Now that the NIEQ has been validated as a psychometric instrument, a direct comparison of NIEQ and DES results using the same participants would be desirable.

## Ethics Statement

This study was carried out in accordance with the recommendations of the UNLV Human Subjects Research Policy of the UNLV Office of Research Integrity, with written informed consent from all subjects. All subjects gave written informed consent in accordance with the Declaration of Helsinki. The protocol was approved by the UNLV Social/Behavioral Sciences Institutional Review Board.

## Author Contributions

CH, VB, JK, DT, and RH: planning. CH, VB, LL-C, JK, DT, and RH: NIEQ design and data collection. SM and RH: analyses. CH, SM, LL-C, AK, and RH: writing.

## Conflict of Interest Statement

The authors declare that the research was conducted in the absence of any commercial or financial relationships that could be construed as a potential conflict of interest.
